# Hunger, Obesity, Public Policies, and Food-Based Dietary Guidelines: A Reflection Considering the Socio-Environmental World Context

**DOI:** 10.3389/fnut.2021.805569

**Published:** 2022-01-18

**Authors:** Alessandra Amorim, Ana de Holanda Barbosa, Paulo José do Amaral Sobral

**Affiliations:** ^1^Department of Food Engineering, Faculty of Animal Science and Food Engineering, University of São Paulo, Pirassununga, Brazil; ^2^Institute for Applied Economic Research, Rio de Janeiro, Brazil; ^3^Food Research Center, University of São Paulo, São Paulo, Brazil

**Keywords:** ultra-processed foods, processed foods, sustainability, food waste, food loss, food classifications

## Abstract

Nowadays, the world has been characterized by hunger, obesity, and food loss and waste (FLW). With the COVID-19 pandemic, the food issue became more intense, serious, and evident. Hunger demands urgent actions. Obesity levels have been raised and are removing health and quality of life from the population. Production planting practices and the food supply chain are not necessarily ecologically friendly. Sustainability issues greatly intensify social problems. As well as food loss (FL), food waste (FW), and sustainability concerns, obesity, and malnutrition are enhanced due to the lack of knowledge by the population. Processed food (PF), packaging, and additives, despite still needing improvement, are essential to food security control. Nowadays, hunger is not due to insufficient agricultural practices but rather to inequality and absence of adequate public policies. In the context of a certain abundance of food production and processing, the hunger scenario in contrast to FLW is an ethical, social, moral, and sustainable issue. In this context, a Food-Based Dietary Guideline (FBDG) can be an important public policy tool from the health, nutrition, environmental, and educational points of view. Despite the effort, the literature shows that FBDGs can be better used to fulfill healthiness and sustainability purposes. In this scenario, the elaboration/revision of the FBDG, adopting a clearer, simpler, and a better-suited communication strategy is essential. In this way, this article discusses the importance of the FBDG as a public policy tool, not only regarding health issues but also communication strategies, production sustainability, and humanitarian ones, which are crucial to FBDG's efficiency.

## Introduction

Past centuries were marked by huge population losses resulting from hunger (1, 2). Nowadays, hunger still exists. According to “The State of Security and Nutrition in the World” report, published by FAO, IFAD, UNICEF, WFP, and WHO (3), around 650 million people suffered from hunger in 2019, representing an increase of 43 million people compared to 2014 and, as a result of the COVID-19 pandemic, it was estimated that around 118 million more people were faced hunger in 2020 than in 2019. By now, this estimation has not been confirmed yet or recalculated. Globally, 149 million children under the age of 5 years were stunted and 45 million were wasted in 2020 (4). Despite the global agreement to eradicate hunger by 2030, the world is off the path to achieve it (3).

At the same time, a greater number of people died as a result of noncommunicable diseases (obesity, diabetes type 2, cancer, etc.) (5–8) and malnutrition (undernutrition—dietary energy deficiency, micronutrient deficiencies, and overweight and obesity—dietary energy surplus) (7, 9, 10). Noncommunicable diseases and malnutrition are considered as the consequences of an unhealthy and unbalanced diet that can include high consumption of processed food (PF).

In 2016, 39% of adults worldwide—which represents 1.9 billion people—were overweight, being that 13% people were obese (11). According to WHO (11), in 2020, 39 million children of 5 years old were overweight or obese. Between the ages 5 and 19, the number was around 340 million in 2016. By 2020, it was predicted that a half of the world's population would be overweight (2). Since 1975, worldwide obesity has nearly tripled (11). Until now, there is no updated obesity statistic from the WHO, considering the COVID-19 pandemic. The readers interested in studying on malnutrition and other diseases issues are invited to consult HLPE report 12 (9), Willett et al. (7), and Swinburn et al. (10).

According to WHO (11), obesity is preventable. Obesity is the excessive fat accumulation on the body, measured by body mass index (BMI), which relates the weight by the square of its height in meters. Values >25 kg/m^2^ indicate overweight and, over 30 kg/m^2^, obesity (11). The WHO (11) explains obesity as a result of an energy imbalance between consumed and expended calories. This imbalance occurs mainly because of the inadequate food consumption—quality, quantity, and frequency—and the sedentary lifestyle—the absence of efficient physical activity (11–13). To change this reality, investments in public policies related to health, agriculture, urban planning, transport, food processing, marketing, and education are essential (11, 14, 15).

The balance between a healthy diet and efficient physical activity is the key point to reduce obesity (11). Nonetheless, Carretero et al. (16) explained that diet is not a product. Diet is the amount of nutrients provided to the body. Each person has individual calories needs according to lifestyle (11, 16). Salt, sugar, and fat intakes have to be restrained; however, their consumption also contributes to improving health. The transport and absorption of soluble vitamins are dependent on the fat in the intestine (17). In addition, adequate oil intake can affect the reproductive feminine system (18).

To avoid obesity, the WHO advise people (11) to limit energy intake from total fats and sugars; increase the consumption of fruit and vegetables, and legumes, whole grains, and nuts; and engage in regular physical activity (60 min a day for children and 150 min spread through the week for adults). Moreover, it advises the food industry for reducing the fat, sugar, and salt content of PFs, ensuring that healthy and nutritious choices are available and affordable to all consumers, restricting marketing of foods rich in sugars, salt, and fats, especially those foods aimed at children and teenagers, and ensuring the availability of healthy food choices and supporting regular physical activity practices in the workplace.

In the modern and globalized world, inefficient and imbalanced diets result in millions of deaths (5–7, 11, 14). Although hunger and obesity must be combated with equal intensity, according to Contreras and Verthein (1), hunger is immoral and more aggressive to health than obesity. According to Sen (19), the food security problem is not only related to food supply chain or food availability but also rather to an entitlement, as a consequence of lack of employment and the absence of good conditions of salary, which are more intense in underdeveloped countries.

The dramatic worsening of world hunger represents a violation of human rights (20). Urgent action and transformation in food systems are needed to ensure food and nutrition security. Public policies aimed to eliminate hunger and poverty are important as food insecurity is the result of political and economic choices. According to FAO, IFAD, UNICEF, WFP, and WHO (3), there are some important pathways toward food system transformation to address the major drivers of food insecurity, malnutrition, and unaffordability of health diets. These pathways are related to: humanitarian and peacebuilding policies in conflict-affected areas; scaling up climate resilience across food system resilience; strengthening the resilience of most vulnerable to economic adversity; intervening along the food supply chains to lower the cost of nutritious foods; tackling poverty and structural inequalities; and strengthening food environments and changing consumer behavior to promote dietary patterns with positive impacts on human health and the environment. Nevertheless, gender inequalities, for instance, must be also considered as the cause and outcome of unsustainable food systems and unequal food access, consumption, and production (21).

Since the Industrial and Green Revolutions, there has been an abundance of food production; however, it was not enough to guarantee food security despite the food industrialization. During the 20th century, according to Aguilera (22), the food industry has shown consistent improvement as a consequence of technological advancements that allowed moving from batch to continuous processing, resulting in the production of thousands of units per hour of microbiologically safe and nutritious food. In addition to food production rising, the food industry development also reduced waste and energy consumption.

The food system inequality and contradiction are a reflection of the lack of public policies and rulers' omission (1, 5, 6). Food is at the core of human health. Having no knowledge or having misinformation about food results in public policy issues (23). The State has a duty to promote society's knowledge, especially when it implies to safety and health (6, 24). Farmers, Food Engineering, Technologists and Scientists, Nutritionists, and Communication professionals can also contribute to humanitarian issues (25). The modern world challenge is, amidst waves of irrelevant information, to promote knowledge and equity to a more demanding and in-need population (2). Thus, clarity is power (2).

Therefore, this review aims to critically discuss the importance of Food-Based Dietary Guidelines (FBDGs) as a public policy tool, not only considering health issues but also considering food production sustainability, humanitarian questions, and communication strategies, which are crucial to FBDG's efficiency.

## Methodology

All the FBDGs presented in this manuscript were consulted on the FAO website (26), in which the link of the original dietary guidelines and the summary with the main information about the document content—such as official name, publication year, stakeholders' involvement, development process, implementation, evaluation, sustainability, and recommendations—for each country are available. The FBDGs written in English, Spanish, or Italian were read by the authors based on the original document, and the documents written in other languages (French, Arabians, etc.) were based on the FAO summary website.

The focus was to identify the strategies adopted on the FBDG construction, especially the ones regarding communication, food classification system, recommendations, and sustainability issues. The nutritional and sustainability analyses were based on the review articles and critical manuscripts developed by the experts in these areas, available on the main research platforms and newspapers, such as Web of Science, Science Direct, Pubmed, and The Lancet Commission.

In addition, the country selection was made with a goal to discuss all the world regions and different cultures (USA, Africa, Europe, Asia, Oceania, and Arabian) to compare the strategies, social, and sustainability concerns, besides the nutritional one.

## Food Supply Management in the Modern World

**Figure 1:**
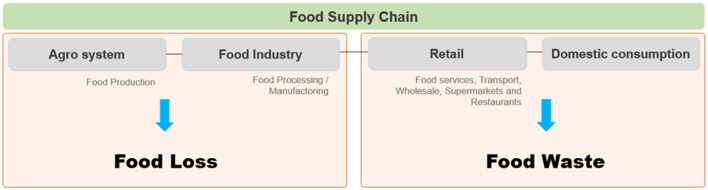
Schema defining food loss (FL) and food waste (FW) in the food supply chain. Teuber and Jensen (28), with adaptions.

Food loss and waste (FLW) are the concepts used to describe losses during the food supply chain management (27–29). Nevertheless, there are divergent definitions about food loss (FL) and food waste (FW) (28). In this article, according to FAO (30), FL will be used to mean losses during the food production and/or processing while FW will be used to mean losses during the retail and domestic consumption. In other words, FL refers to losses at pre- and post-harvest, during the harvest, and food processing and FW refers to losses of food destined to human consumption (Figure 1) (27, 29).

In addition to intensifying a clear contradiction between food production and hunger, reducing FLW is an urgent need to improve sustainability (28, 30). FLW imply in the misuse of the world's limited energy. It represents an inefficient use of natural resources (particularly water and land) and a useless greenhouse gas emission (29), representing an evitable and unnecessary environmental impact (31). The world is going to have around nine billion people in the near future (32), thus, to feed this population, natural resources must be preserved.

Furthermore, adequate food is a universal, constitutional, and multidimensional human right advocated by the United Nations (UN) (33). Into sustainability dimension, this right is part of the duty to guarantee quality food access in sufficient quantities in the long term. Presently, at least 31% of the world's food production was lost or wasted, meaning that, around 1.3 billion tons of food have been wasted (1, 27, 34–36). According to Sharma et al. (35), the FLW represent 1.4 billion ha of fertile land, which encompasses 28% of the world agricultural area, 3.3 billion tons of CO_2_ equivalents, and USD 936 billion undermanaged. Reducing FLW can represent USD one trillion in terms of the economy (29).

Food loss and waste occur during the food supply chain, from harvesting to home consumption (1, 34, 35). Between 25 and 40% of the vegetable production is lost due to the hygienic-sanitary conditions or because of a lack of standardization of quality criteria (size, color, texture, shape, appearance, etc.) or even due to the absence of an efficient cold chain (1). Moreover, 45% of the fruit and vegetables produced worldwide are not consumed, which corresponds to USD 2.6 trillion literally thrown in the trash, if social and economic aspects are considered (35). To reduce this impact, pesticides and synthetic fertilizers allowed by the Food Administrations and Agencies of each country are usually used. However, this method of reducing FL can imply in chemical contaminations harmful to the environment and to the customer's health (1).

On the other hand, according to Flanagan and Priyadarshini (34), 30% of the FW occurs during home consumption and 10% in the retail segment. Between 40 and 60% of all the household waste is FW, and 2/3 of the FW in Europe is avoidable. Furthermore, Flanagan and Priyadarshini (34) estimated that 50% of the human food production is wasted. Between 3 and 10% of the FW can result from inappropriate storing conditions besides labeling and shelf-life misunderstandings.

The population do not necessarily have knowledge about sustainability issues, FLW, and which kind of action they must take to reduce FW. For example, according to Williams et al. (31), UK consumers are more concerned about the discarding of the packaging than FW because they associate discarded packaging with environmental issues. Nevertheless, FW provokes huge environmental problems in underdeveloped countries. Moreover, there is a contradiction between sustainability discourse and consumption practices (37). In Brazil, routine habits such as eating, cooking, cleaning, and personal care are not sustainable (37), and they include FW resulting from exaggerated foodstuffs purchase (36). Some cultures—such as North-American and Brazilian—understand abundance as social growth and it directly implies (un)sustainable consequences (37, 38). Moreover, domestic and routine habits are cultural and unconscious habits that follow moral and belonging rules (37).

The packaging system and industrialized food are criticized because of sustainability issues. It is true that some packaging and food production systems are not ecologically friendly yet (such as meat production and nonbiodegradable polymers). Moreover, there are chemical components applied in food production and food processes that can be harmful for health (1, 39). However, most of the additives applied in the food industry are healthy and from natural sources (40–42). These additives are an important element to raise shelf-life and, consequently, reduce FW and hunger (40). As well as packaging, additives can be considered as more beneficial than damaging to the planet. In addition, scientists around the world are doing their duty to improve industry sustainability and develop a sustainable food supply system, such as emerging technology, circular economy, bioeconomy, and environmentally friendly packaging, among other approaches.

In a context of the certain abundance of food production and processing, the hunger scenario in contrast to FLW is an ethical, social, moral, and sustainability issue (34, 36). Unlike the past centuries, hunger is not due to insufficient planting, but rather to the inequality and absence of adequate public policies (1, 5, 6), and FLW is even part of the Sustainable Development Goals (SDGs) of the UN (27, 43). Moraes et al. (29) described that government authorities have the role to advocate at all levels to implement sustainable programs. In this way, the FBDG can be a powerful tool.

## Food Classification System

Currently, to improve health as well as to reduce noncommunicable diseases and malnutrition, more than a hundred countries worldwide developed FBDGs to orient their population in their food choices and healthy lifestyle. By classifying food and guiding people about the quantity and frequency of food intake, the FBDG also contributes to the achievements of SDGs (44, 45). The FBDG recommendations can indirectly influence the amount of CO_2_ emitted during the food production (15, 44, 46). Nonetheless, according to Ritchie et al. (44), the food intake and food classification recommendations are not clear. Therefore, to achieve the success of SDGs, an FBDG review is essential.

Food-Based Dietary Guideline is a public policy tool used by rulers—especially health authorities—to communicate and educate their population about food choices. The Italian FBDG highlights that this document must consider the environmental characterization, in other words, the FBDG must be appropriated to the economic, geopolitical, physical (availability), and sociocultural context (47). In this way, a multidisciplinary committee composed of technicians and scientists must elaborate the FBDG. Generally, health scientists (mainly nutritionists and medical doctors) elaborated food classifications (26).

Most of the FBDG used food classification by their nutritional composition (Table 1). The North American FBDG classified food as vegetables, fruits, grains, proteins, and dairy (48). The Spanish one, in turn, classified food as wholegrain cereals and products, fruits, vegetables, olive oil, dairy products, fish, poultry, pulses, nuts, potatoes, eggs, red meat and meat products, sweets, snacks, and sweetened beverages (49). On the other hand, the Brazilian FBDG classified food according to what the authors considered as being food-processing levels (50). Moreover, in turn, the Uruguayan FBDG classified foods by their nutritional composition (vegetables and legumes; fruits; breads, flour, pasta, rice, and potatoes; milk and cheese; meat, fish, and eggs; seeds and oils; and sugars and sweets) and, inside these groups, distinguished them by their processing level (51) (Table 1).

**Table 1:** FBDG communication strategies relating to food classification.

**Country**	**Is the communication strategy graphic-visual?**	**Is the food classification system according to**	
		**Nutritional composition?**	**Processing level?**
Spain	•	•	
Italy		•	
France	•	•	
Portugal	•	•	
UK	•	•	
USA	•	•	
Canada	•	•	
Brazil			•
Argentina	•	•	
Chile	•	•	
Uruguay	•	•	•
Ecuador	•	•	
South Africa	•	•	
Australia	•	•	
United Arab Emirates	•	•	
India	•	•	
China	•	•	
Japan	•	•	

*FAO (26)*.

Currently, at least seven food system classifications by processing levels are known (Table 2). According to the Scientific Committee of the Spanish Agency for Food Safety and Nutrition (52), they are useful as complementary epidemiologic studies, such as the prevalence of obesity in specific geographical areas according to specific population groups—child, indigenous, and breastfeeding, for example—in economically disadvantaged sectors. Talens et al. (52) explain that each one of those classifications has its own definition of process and the classification coverage can be local (IFIC, UNC, NIPH, and IFPRI) or global (NOVA and SIGA). In addition, the readers interested in understanding more about these food classifications are invited to consult (52).

**Table 2:** Food system classifications according to processing level.

**Name**	**Location**	**Classification system**
IARC-EPIC	Europe	1- Not processed food 2- Minimally processed food (industrialized or housewifely) 3- Industrialized food
IFIC	USA	1- Minimally processed food 2- Processed food by simple conservation 3- Processed food 4- Convenience food 5- Packaged food
UNC	USA	1- Not processed or Minimally processed food 2- Processed food–simple level 3- Processed food–moderated level 4- Processed food–intense level
NIPH	Mexico	1- Modern industrialized food 2- Traditional industrialized food 3- Home processed food
IFPRI	Guatemala	1- Not processed 2- Minimally or Partially processed food 3- Highly Processed food
NOVA	Brazil	1- No processed or Minimally processed food 2- Culinary ingredients 3- Processed food 4- Ultra-processed food
SIGA	France	1- Not processed or Minimally processed food (A_0_, A_1_, and A_2_) 2- Processed food (B_1_ and B_2_) 3- Ultra-processed food (C_1_, C_2_, and C_3_)

*Talens et al. (52)*.

For being elaborated by health professionals, none of these classifications followed the definition of food processing as described by the Food Science, Technology and Engineering (FSTE) (53). Despite them being a processing level classification, most of these classifications were defined according to the food ingredients (52). Furthermore, only NOVA classification, whose description can be found in Moubarac et al. (54) and Monteiro et al. (55), was applied in an FBDG.

From the FSTE point of view, *minimally processed food* (MPF) is washed, sanitized, cut, or chopped foods, which are microbiologically safe and stable under the convenient packaging system, and are not thermally treated (56), while PF is a food product obtained by a sequence of unit operations (23, 57), usually different from these related to MPF and with an important energy footprint (53). Overall, MPF is important because it is practical to use and can reduce cooking time and reduce FW at home, as these foods have been cleaned and cut, and their seeds and husks eliminated in the industry. And, PF is important mainly because of its long shelf-life, which means that the consumer can eat these foods several days or even months after-acquired. Nevertheless, overall, PF has also a negative appeal. However, the benefits of food processing must be recognized. For example, the benefits of food processing by thermal treatments include the inactivation of foodborne pathogens, natural toxins, or other detrimental constituents, prolongation of shelf life, improvement of digestibility and bioavailability of nutrients, improvement of palatability, taste, texture, and flavor, and enhancing functional properties, including augmented antioxidants and other defensive reactivity or increased antimicrobial effectiveness (58), besides contributing to decrease FLW. More definitions of PF foods and unit operations for food processing can be found in Jones (59), Floros et al. (23), and Aguilera (60).

Furthermore, recently, scientists from Sorbonne University (France) developed a nutritional system classification, called Nutri-Score (or 5C). Easy to understand, due to the adopted visual communication strategy, the Nutri-Score classification can be applied in food labeling (61). Nutri-Score classified food into five groups (A, B, C, D, and E) according to the food nutritional value, decreased from A to E (61, 62). The Nutri-Score classification system is used by the French and Spanish Health Ministry (61, 63). Botelho et al. (57) reinforced that, to identify the real source of nutrients, it is indispensable to examine food group classification. Furthermore, besides the FBDG use, food classification is also a strategic tool for epidemiological studies and health treatments.

## FBDG Role

Food-Based Dietary Guideline has been used as a tool to improve health and sustainability worldwide (13, 43, 59, 64–67). As a strategy to achieve the UN's 17 goals, the SDGs also include clear and correct communication about nutrients and diet (16, 43).

Proposing solutions to feeding issues is complex (23, 68). It involves social, cultural, economic, and moral issues besides requiring a technical multidisciplinary knowledge. Generally, health professionals are involved in FBDG development (26). These professionals are experts in understanding how the ingredients and their nutrients are metabolized by the human body, representing something beneficial or not to health according to their frequency and quantity intake. This analysis is important to the success of FBDG; nonetheless, it is not enough to achieve its purpose.

Besides the nutritional point of view, these guidelines should also consider the fact that the target population, and the society as a whole, is made up of individuals who interact with each other. At most of the time, these same individuals respond to incentives and face trade-offs. Therefore, what is expected is that FBDG and/or policymakers have the knowledge of an optimal allocation of resources in the economy for consumers, producers, and the food system.

It should be noted that consumer demand for food is an important element in the formulation of several agricultural and food policies. Changes in food prices and income are the determinants of food demands. As Blundell (69) stated for some policy issues, the importance of empirical evidence on consumer behavior is indisputable. Price and income demand elasticities for food inform policymakers and researchers about how consumers make food purchasing decisions and help the design of effective nutrition policies.

It is a recurrent empirical finding, in several countries and at different historical moments that the participation of food expenditures in the family budget decreases as their income rises. In fact, this is one of the most established empirical findings and regularities in economics and is known as “Engel's law,” due to the studies by Engel (70). The reference for the validity of “Engel's law” is Houthakker (71), but Chai and Moneta (72) can be also consulted for a useful retrospective on Engel's work. Moreover, Chattopadhyay et al. (73) used Engel's law to develop a mathematical model that can be applied as a tool for economic policy formulation. In addition, Lancaster (74) proposed an alternative view on the consumer theory, that the goods are, in fact, a collection of characteristics. Sen (75), in turn, includes the functionality attributed by the person to the goods on the Lancaster consumer theory: In Sen's terminology, a “functioning” is what an individual chooses to do or to be, in contrast to a commodity, which is an instrument enabling her to achieve different functioning. Sen (75) states that it is not merely the achieved functioning that matters but the freedom that a person has in choosing from the set of feasible functioning, which is referred to as the person's “capability.” This has become the so-called capability approach. This approach has been immensely useful in the context of studying poverty, gender issues, political freedom, and the standard of living (76).

Engel's law has two broader implications for the structure of consumption expenditure (77). First, there is a tendency to food specialization of the poorer's budgets in the sense that they are less diversified than those of more affluent consumers. Within the food budget, cheaper, more starchy foods (such as rice, potatoes, and bread) are likely to be predominant for the poor, leading to less nutritious, less diversified diets (77). The second implication of Engel's law is related to the quality of consumption. The declining food share that accompanies income growth means that the quality of consumption rises. Moreover, as food is the good consumed intensively by the poor, there is a natural link between Engel's law and the measurement of quality. Based on a study for more than 150 countries, Clements and Si (77) found out some interesting relations between Engel's law, the variety of foods in the diet, and their quality. While the food share falls with higher incomes, there is a tendency for spending to be more evenly over foodstuffs reflecting a more diverse diet.

Diet, economic-social matters, and lifestyle are linked. Therefore, to achieve the purpose of FBDG, a multidisciplinary technical body is needed, and it includes social, economic, and human food chain (HFC) professionals. Health professional knowledge is part of the HFC. However, the HFC embraces soil handling, food production system, the complex and extensive food processing, filling and packaging, storage conditions at the sale point, and consumption. Thus, in addition to health professionals, the HFC must also be studied by Agronomists, Food Engineers, etc. Efficient actions toward better health standards applied in public policies demand interdisciplinary strategies, with public–private and academic support (5, 14, 23, 68).

Some countries used different strategies in their FBDG, in some cases, including the target audience, such as the general population, breastfeeding, and children to the age 2, eldering, and indigenous (13, 65, 66, 78, 79). Generally, FBDGs encourage the consumption of water and a diversity of food in different proportions, always associated with regular physical activities (13, 65, 66). Ingredients such as sugar, fats, and salt are shown as items to be avoided or limited (13, 26, 65, 66). Among all the FBDG presented in Table 1, only those from Brazil and Canada do not recommend the regular practice of physical activities. In June 2021, the Brazilian Health Ministry released the “Physical Activity Guide for Brazilian Population” in a complement to FBDG. All FBDG can be found on the FAO website (26).

French, Chilean, and South African FBDG recommend the consumption of food rich in starch daily as a food base. According to Herforth et al. (65), more than half of the 90 FBDG analyzed in her review also encourage it. The UK FBDG, in turn, recommended several sources of carbohydrates as a food base, including bread and pasta. The South African FBDG focusing on regional foods was elaborated.

The Spanish FBDG—the healthiest country in the world, according to Bloomberg Global Health Index 2020 (80)— opted for a visual communication strategy, in which combinations of physical activities and food choices were suggested, specifying quantities and frequencies. To Portuguese, Argentinian, and Chinese FBDG, healthy feeding should be complete, balanced, varied, and followed by physical activity. Italy—the second healthiest country in the world according to Bloomberg Global Health Index 2020 (80)— developed a technical FBDG explaining some “true or false” food issues, clearly and straightforwardly. Chile's FBDG, in turn, recommends reducing the TV time and increasing the fast walking. USA's FBDG explains that the energy intake should be appropriated by the personal needs. In addition, the North American FBDG highlights that the food choice must respect the individual preferences and cultural habits.

Italian, French, Argentinian, Australian, Chinese, and Indian FBDG, besides the reduction of salt, sugar, and fat intake, also recommend to limit the consumption of alcohol. Brazilian, Canadian, Indian, Uruguayan, Ecuadorians, and Australian FBDG extend this recommendation to PF (Brazilian, Uruguayan, and Ecuadorian FBDG— “ultra-processed” food and, Canadian and Australian FBDG— “highly processed” food), whereas British and Indian FBDG extend to tabaco. Indian FBDG recommends limiting PF consumption, however, the distinction between industrialized food (PF) and fast food (restaurants franchise) is not clear. Ecuadorian FBDG, in turn, encouraged the reduction of PF, fast food, and sweetened beverages, as the Brazilian one. Among all FBDG presented in Table 1, Japanese FBDG was the only one to orient their population to reduce FW. This is remarkable!

The majority of the countries adopted the visual communication strategy, except Brazil and Italy (13, 65, 66, 79). Up to date, Italian visual communication has not been presented (26). Canada^1^, USA,^2^ and France^3^ also use a website to communicate with the population. According to Hess et al. (67), visual communication strategies do not change the FBDG efficiency and efficacy if the information is easy to understand and to follow.

To be effective, besides culturally accepted, the message must be clear, concise, practical, accessible, and easy to be remembered (59, 65, 78, 79). The United Arab Emirates FBDG used a tourist/architectural-cultural landmark of the country as a visual communication strategy, the “Burj Khalifa”. This structure represents the feeding. The base of the structure is water. Each color represents a food group (cereals, vegetables, dairy, fruit, meat, and fat), and its proportion represents the quantity/frequency of the consumption (26). The Japanese FBDG applies a similar strategy (a popular toy).

There is a lack of data on the literature about the FBDG effectiveness. In the USA, according to Floros et al. (23), the FBDG implementation prompted companies to change the product's formulation and to create foods that are more nutritious. Baked products and cereals now have higher fiber content and use whole grains. Convenience-store food made of fruits, vegetables, and whole grains became available at the markets. The baby carrot, not existent as of then, was widely accepted by the target audience. After reformulations, the trans-fat content was reduced in many products (23).

All the FBDG shown in Table 1 classified food according to their nutritional composition, with an exception of the Brazilian FBDG (52). The Uruguayan FBDG classified food according to their nutritional composition and, inside each group indicated the processing level as well. In his strategy, the Brazilian FBDG classified food by their processing level based on the NOVA classification (50). Nonetheless, the main criterion on the NOVA classification is not necessarily linked to process as the action of processing food—using a sequence of unit operations—but according to the ingredients used in the formulation of the food, in other words, the product or chemical component added pre-, during, or post-processing.

In the NOVA classification, the term “ultra-processed” food stands out, which was associated with products with a low nutritional value (8, 16, 52, 62, 81). According to Monteiro et al. (55), “ultra-processed” food would be an industrial formulation with an additive not used in domestic cooking (8, 81). Nevertheless, many Chefs are also using ingredients that are rarely used at home (40), but they are not considered as being “ultra-processed” food producers. Indeed, it is not so easy to define UPF because it can be so heterogeneous in nutritional composition, as demonstrated by Lorenzoni et al. (82), thus representing a heterogeneous group of foods with different characteristics.

Furthermore, according to Gibney (83), there was no official definition of the “ultra-processed” term, and the way that the author used it has changed over the years (16, 32, 52). Canada and Australia's FBDG do not use this strategy and also recommend avoiding “high processed” products, which are defined by them as products with high salt, sugar, and fat content; different from the “ultra-processed” definition.

According to the Scientific Committee of the Spanish Agency for Food Safety and Nutrition (52), there is no relation between health and the type or intensity of processing level (57, 84). Nutritional quality and (ultra)processing are distinct concepts that can affect health in different ways by their own mechanisms (62). Nutritional value is related to the food formulation or composition (57), regardless of whether it is made at home, restaurant, or industry. Petrus et al. (53), Carretero et al. (16), and Knorr and Watzke (81) related that the argumentative basis of NOVA classification is ingredients and not process parameters. Adding ingredients is part of the formulation (57) and it is not related to process parameters. Process parameters arguments must involve temperature, pressure, time, amount or flow rate (for noncontinuous or continuous processes), and others, not ingredients. At home and in restaurants as well, homeworkers and Chefs also freeze, refrigerate, cook, ground, mold, dry, fry, and apply other unit operations (60). This made the Brazilian classification (NOVA) not comprehensible, accessible, practical, or viable (16, 52, 59, 84).

Knorr and Watzke (81), Derbyshire (85), and Petrus et al. (53) considered the term “ultra-processed” more misleading than explanatory. Sadler et al. (84), Carretero et al. (16), Jones (59), and Talens et al. (52) reported that diets lacking “ultra-processed” food could also exceed the recommended amount of calories. In Brazil, salt and sugar intake is higher in food made at home than in industrialized ones (53). Ares et al. (8) described that the term “ultra-processed” is not widely understood. Galan et al. (62) showed that 21% of the ultra-processed food classified by NOVA has good nutritional quality. In addition, Petrus et al. (53) remind that the NOVA classification encourages raw or unprocessed food consumption, which cannot be safe and can increase foodborne diseases. Therefore, the NOVA classification and the “ultra-processed” term do not necessarily contribute to achieve healthily the SDGs. Consequently, the Brazilian FBDG should be revised in terms of its food classification system adopted.

## Future Challenges

Obesity is not an individual responsibility factor due to mistaken motivational choices (23, 86). Obesity and malnutrition can also be related to sustainability issues (7, 12, 86). The global warming consequences in food production will affect the underdeveloped countries more intensely, especially their economically disadvantaged part. According to Kleinert and Horton (86), solving malnutrition and obesity is necessary to implement sustainable business models with a focus on health promotion. It is not only enough to produce quality food but also self-sustainable and accessible food to the population.

The COVID-19 pandemic has shown that the current accessibility and production food system have not been efficient in protecting the population against hunger and obesity. In a social, economic, and health crisis scenario, we saw—at the same time—increasing hunger, obesity, and FLW (87). Not surprisingly, the 2020 Nobel Peace Prize was awarded to the UN's World Food Program. According to Berit Reiss-Andersen, chair of the Norwegian Nobel Committee, food access cannot become a weapon of war and conflict (88).

The current global food system not only fails in fulfilling the basic nutritional needs but also intensifies the pressure on the planet's sourcing boundaries (15, 44). According to Earth Overshoot Day (89), a metric used to identify the point (in days) when humanity's demand for ecological resources exceeds what the Earth can regenerate at the same year, the Overshoot Day 2020 happened on August 22, 2021 and on July 29, 2021. The carbon footprint increased 6.6% from 2020. In other words, almost half of the planet resource consumption in 2021 will not be recovered in the same year.

According to Springmann et al. (45), the FBDG can also play a strong role in sustainability issues, which has not been adequately explored. FBDG, by guiding what and how much to eat, indirectly influences the amount of CO_2_ generated during the food supply chain (15, 44–46). Nevertheless, Ritchie et al. (44) report a lack of clarity in the recommendations.

In the future decades, the increasing population, urbanization, and globalization will pressurize the world, whereas natural sources will be increasingly scarce (14). Besides sustainability issues, it is worth noting one aspect regarding eating habits. In most western countries and based on women's increasing participation in the workforce, the food away from home (FAFH) is an increasing trend component of total food consumption and nutritional intake of adults and children. Empirical evidence shows that FAFH has been associated with poor diet quality (90, 91). Hence, policies designed to influence nutritional and healthy outcomes would be incomplete if they did not address the role of FAFH (92).

In relation to home production (unpaid domestic and care work), it is interesting to note that, mostly women, home cooking declined in the late century and in the early years of the 21st century (93–96). Historically, food preparation and household cooking have been assigned to women, and food at home (FAH) has been linked to female gender roles and identity.

Women have also had an important performance in the traditional food knowledge (TFK). The TFK refers to a cultural tradition of sharing food, recipes and cooking skills, and techniques and passing down that collective wisdom through generations (97). According to Kwik (97), the value of this knowledge is hidden in a global food system offering an abundance of commercial convenience foods, which is a consequence of urbanization and is intensified by a dynamic lifestyle (98). In addition, in their study with children in the Netherlands, Folkvord et al. (99) explain that food exposition as the cooking programs on TV can influence eating behaviors. On the other side, according to Contreras and Ribas (100), the “omnivore's deculturalization” is not related to food industrialization but rather to food medicalization.

Although men have increased their contribution to home cooking (94), gender division of labor remains unequal, with women doing most of the household chores. In most societies, women keep carrying the responsibility for labor of food provision—the most basic labor of care. Another interesting topic to point out is related to the elderly or the ones who retire. Based on what was noted by Becker (101), that consumption is the output of a “home production” function that uses both expenditure and time as inputs. Aguiar and Hurst (102) were the first ones to address the topic of meal preparation. They recognize the inputs of food production include not only just food (modeled by food expenditures) but also the time spent shopping and preparing meals. They also showed that despite a sharp decline in food expenditures, neither the quantity nor the quality of food intake deteriorates with the retirement status. Also, what they find is that these declining expenditures are offset by increased time spent shopping and preparing meals, suggesting that time and money are substitutes in food production. Nevertheless, these practices are not necessarily defined only by prices/expenditure (some monetary measure) and time. Then, better FBDG outcomes would require other considerations, which are multiple (nutritional, environmental, social, and also economic among others) and varied.

Thereby, while the food production system does not pay attention to environmental, nutritional, social, and economic issues, no other measure will be efficient (15). The food production system begins in the cultivation technique, passing through processing food, filling, distribution to the market, and storage to provide effects on the human body. Despite an unquestionable technological development, while ensuring the scale production of microbiologically safe, nutritious, and appealing foods, the industry must also engage consumers and its stakeholders as well (103).

The food production chain will be sustainable to the planet and to the individuals only when the public–private partnership and academia are strongly established (Agronomic Engineering, Food Engineering, Health, and Public Policies) starting with a clear and an educational FBDG elaboration. In addition, the food industry must increase its transparency. A critical review of the abovementioned issues is essential for achieving the SDGs.

## Conclusion

Malnutrition and obesity are the consequences of imbalance and inequality diet. Currently, with a certain abundance of food as a consequence of the food production and the food industry, the accessibility of food quality and balanced food consumption emerged as a new concern, both intensified by the absence of the population knowledge. In the contemporary world, malnutrition exists because of the inefficiency of public policies, social inequality, low purchasing power, and poor industrial-governmental agreements. Obesity is a preventable biopsychosocial and environmental pandemic, resulting from unhealthy lifestyles in a technological, sedentary, and urbane system. In this contradiction, the under-management resources are evidenced by the nonsustainable food supply chain practices, with high levels of FLW, which result in overload of the planet and rising food insecurity. To aggravate this situation, the daily population habits are not sustainable, most of the time, made unconsciously, and in 2020, the world was affected by the COVID-19 pandemic that challenged the social, structural, and ecological world system.

Because of this scenario already existing and serious even before the COVID-19 pandemic, governments worldwide developed FBDGs with a goal to orient and educate the population in their food choices and, in consequence, in sustainability issues as well. The FBDG should inform the population about the current problems and orient their decision-making to mitigate them. However, this important public policy tool can and should be better used from a healthy, nutritional, social, and environmental point of view. Some FBDG, especially the Brazilian and the Uruguayan ones, choose an incorrect and misunderstood food system classification (NOVA classification) in terms of the process definition. FBDG must be clear, correct, and practical, otherwise, it will confuse the population and therefore lose its purpose and distort the economy.

With the COVID-19 pandemic, hunger, malnutrition, obesity, and FLW have been intensified. Evidence shows that we are not on the path to achieve the goals of SDGs. Moreover, we are facing a dramatic transformation in our access to and the availability of food—along with where we eat and with who. Therefore, a radical change in the feeding system is urgent and necessary.

## Author Contributions

AA contributed to conceptualization, research, writing—original draft, editing, and reviewing. AB contributed to research and writing and reviewing. PS contributed to writing and reviewing, supervision, and project administration. All authors contributed to the article and approved the submitted version.

## Funding

The São Paulo State Research Foundation (FAPESP), for the grant (2013/07914-8) and the Brazilian National Council for Scientific and Technological Development (CNPq), for the grant of ALH (44.3196/2019-2), and research fellowship of PJAS (30.0799/2013-6). This study was financed in part by the Coordenação de Aperfeiçoamento de Pessoal de Nível Superior—Brasil (CAPES): Finance Code 001 (MS fellowship of AA).

## Conflict of Interest

The authors declare that the research was conducted in the absence of any commercial or financial relationships that could be construed as a potential conflict of interest.

## Publisher's Note

All claims expressed in this article are solely those of the authors and do not necessarily represent those of their affiliated organizations, or those of the publisher, the editors and the reviewers. Any product that may be evaluated in this article, or claim that may be made by its manufacturer, is not guaranteed or endorsed by the publisher.

## Footnotes

^1^ https://food-guide.canada.ca/en/

^2^ https://www.myplate.gov/

^3^ https://www.mangerbouger.fr/
